# Correlation of quantitative computed tomography derived bone density values with Hounsfield units of a contrast medium computed tomography in 98 thoraco-lumbar vertebral bodies

**DOI:** 10.1007/s00402-021-04184-5

**Published:** 2021-09-25

**Authors:** Fabian Buenger, Yasser Sakr, Niklas Eckardt, Christian Senft, Falko Schwarz

**Affiliations:** 1 Department of Neurosurgery, Jena University Hospital-Friedrich Schiller University Jena, Jena, Germany; 2 Department for Anaesthesiology and Intensive Care, Jena University Hospital-Friedrich Schiller University Jena, Jena, Germany; 3 Department for Radiology, Jena University Hospital-Friedrich Schiller University Jena, Jena, Germany

**Keywords:** Hounsfield units, Bone mineral density, Quantitative computed tomography, Lumbar spine, Contrast medium

## Abstract

**Introduction:**

Vertebral fractures in patients with bone density reduction are often a major challenge for the surgeon, as reduced bone density can lead to screw loosening. Several options are available to determine bone density preoperatively. In our study, we investigated the correlation of Hounsfield units (HU) of a contrast medium computed tomography (CT) to the bone density values of a quantitative computed tomography (QCT) and computed a formula to estimate bone density values using HU.

**Materials and methods:**

In our retrospective data analysis, we examine 98 vertebral bodies from 35 patients who received a contrast medium CT of the spine and a QCT, performed no longer than 1 month apart. The determined HU from the contrast medium CT were compared with the bone density values of the QCT and examined for correlations. Linear logistic regression was used to estimate bone density values base on HU.

**Results:**

A strong correlation was found between the HU measured in the CT and the bone density values (*r* = 0.894, *p* < 0.001), irrespective of patients’ gender. We also found no correlation differences when the HU were measured at different levels. Bland–Altman plot demonstrated good agreement between the two measurements. The following formula was developed to estimate bone density values using HU: QCT-value = 0.71 × HU + 13.82.

**Conclusions:**

Bone density values correlate well to HU measured in contrast medium CT. Using simple formula, the bone density of a contrast medium CT of vertebral bodies can be estimated based on HU without additional examinations and unnecessary costs.

## Introduction

Knowledge about bone density is essential for preoperative planning in patients with vertebral body fractures [1]. For optimum treatment results, one must not overlook an underlying osteoporosis. Due to demographic trends, an increasing proportion of elderly patients, who frequently suffer from reduced bone density are to be expected in the next years [2, 3].

Quantitative computed tomography (QCT) has been routinely used to determine bone density. Recently, there have also been an increasing number of publications that determine bone density using the Hounsfield units (HU) in a native computed tomography (CT) [4, 5]. In our own study, we were thus able to create a formula for patients with a native CT [6].

In the present study, we investigated the possible correlation between HU and QCT values in patients who received a thoraco-lumbar CT with contrast medium. Further, we aimed at developing a formula to calculate bone density based on the contrast medium computed tomography alone, thus possibly rendering additional QCTs redundant.

## Materials and methods

We performed a retrospective data analysis of 98 vertebral bodies of 35 trauma patients who received contrast medium CT of the thoraco-lumbar spine and QCT of the lumbar spine in our department between 1 January 2015 and 15 February 2019.

All data were collected and processed using IBM SPSS statistical program for Windows (version 23; SPSS, Inc., Chicago, IL, USA). The following factors were determined: age, sex, mean values of Hounsfield units, mean values of QCT, date of each examination. The study was approved by our local ethics committee.

### Inclusion and exclusion criteria

All patients who received CT with contrast medium including the lumbar spine as part of a polytrauma CT scan were included. The axial, coronal and sagittal planes of the vertebral bodies had to have been reconstructed. QCT had to have been performed within 1 month prior or after. HU were measured in contrast medium CT from the 11th thoracic vertebral body to the 4th lumbar vertebral body and compared with the corresponding values from QCT of the associated vertebral bodies.

If pathologies, e.g. tumour, haemangioma, fractures, implants, cement augmented vertebral bodies, spondylodiscitis or osteochondrosis were detected in the CT, the respective vertebral bodies were not used for the measurements.

### Measurement of the Hounsfield units

**Fig. 1:**
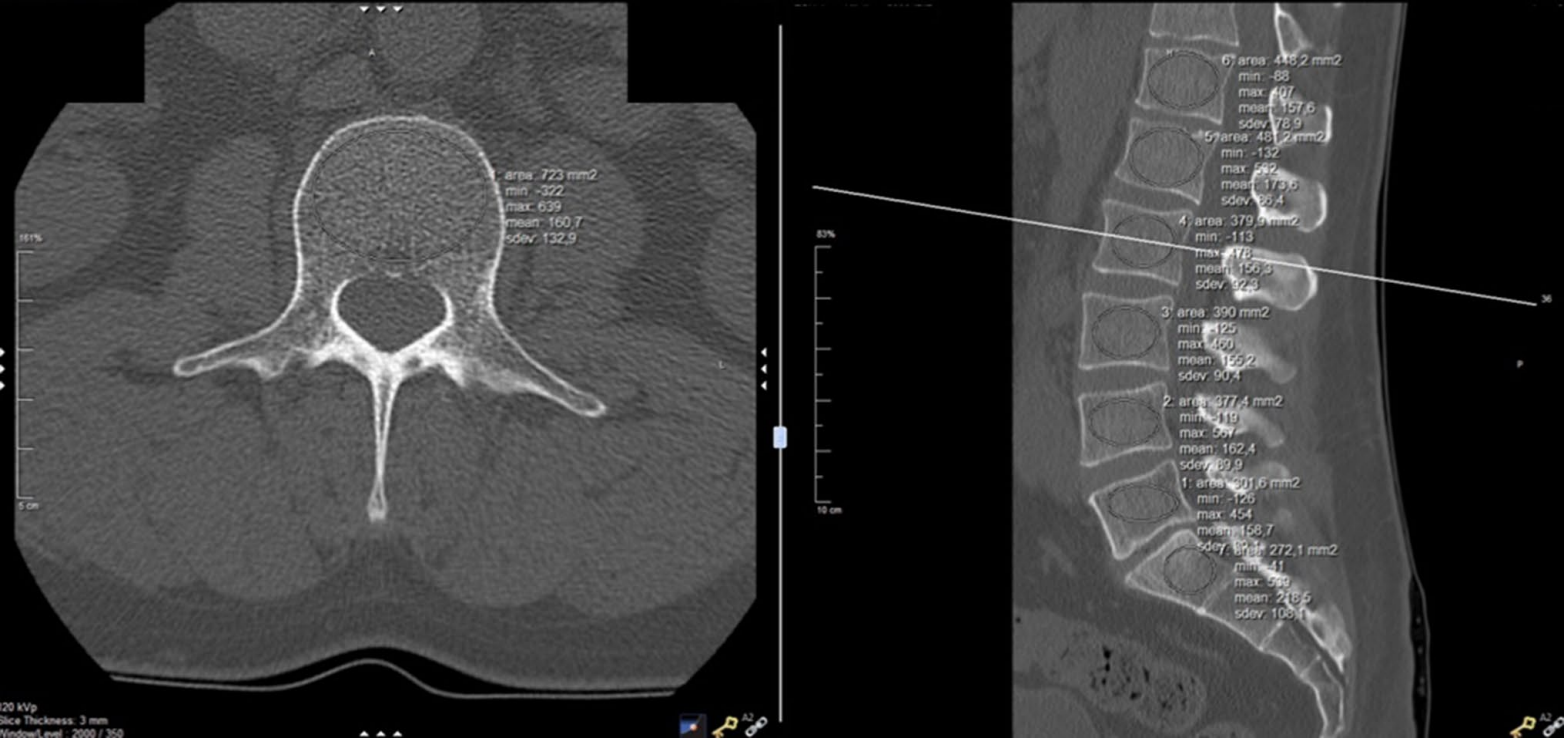
Computed tomography of the spine with axial (left), sagittal (right) measurement of the Hounsfield units using an elliptical measurement field in the center of the vertebral bodies

Measurements were performed using CT (General Electric Medical Systems, USA, Revolution) in axial, sagittal, and coronal planes. The measurement field was chosen to be as large as possible, leaving out the cortex as described previously [6] (Fig. 1).

HU were determined with the software CernerSkyVue PACS Image Viewer (Cerner Deutschland GmbH), using the “Mean” value for further calculation. The mean value was determined from each plane and the mean HU was calculated from this. The measurements were always taken by the same physician.

### Measurement of the quantitative computed tomography values

QCT values were measured on a CT scanner (General Electric Medical Systems, USA, Revolution EVO). The calibration phantom consisted of K_2_HPO_4_ and was placed under the patient. Bone density was determined using bone mineral densitometry software (QCT-Pro™, version 3.1, Mindways Software).

### Statistics

Categorical data are given as number (*n*) and percent (%) and continuous data as mean with standard deviation (SD) or median with interquartile range (IQ).

Pearson’s correlation coefficient or Spearman-Rho was used to use the correlation of 2 variables. A value of *r* = 1 indicated that there was a linear relationship. A value of *r* = − 1 showed an inverse correlation of the variables. A *t* test was used to test whether the determined correlation coefficients differ from zero. Bland–Altman plot was used to assess the agreement between measurements. Linear logistic regression was used to estimate QCT values from HU units. A significance was evaluated as a value below 5%.

## Results

At the time of examination, the median age of the patients was 62 years (IQ: 53–74). Measurements were taken on 71 vertebral bodies of male (72.4%) and 27 vertebral bodies of female patients (27.6%). The median interval between contrast medium CT and QCT examination was 3 days (IQ: 1–7). The 2nd lumbar was the most frequently examined vertebra with 30 measurements. Details are provided in Table 1.

**Table 1:** QCT-values and the corresponding Hounsfield units according to the different vertebras

	Count	QCT-values^a^	Hounsfield units
Th 11	5	105.8 (76.7–111.9)	114 (105.3–151.2)
Th 12	5	101.8 (82.9–134.7)	119 (109.4–176.2)
L 1	18	115.6 (80–133)	157.5 (95.2–176.6)
L 2	30	99.1 (57.6–135.2)	134.2 (98.7–165.9)
L 3	25	102.3 (60.2–124)	131.6 (93.3–164.3)
L 4	15	96.3 (67.8–132.4)	107.7 (94.6–107.7)
All levels	98	103.5 (85.9–128.9)	131.5 (98.4–164.4)

Values are presented as median (25–75% interquartile range)

*L* lumbar, *Th* thoracic

^a^mg/cm^3^ K_2_HPO_4_

The median HU of all vertebral bodies was 131.53 (IQ: 98.4–164.38), and 103.5 mg/cm^3^ K_2_HPO_4_ (IQ: 85.9–128.9) for QCT, respectively.

**Fig. 2:**
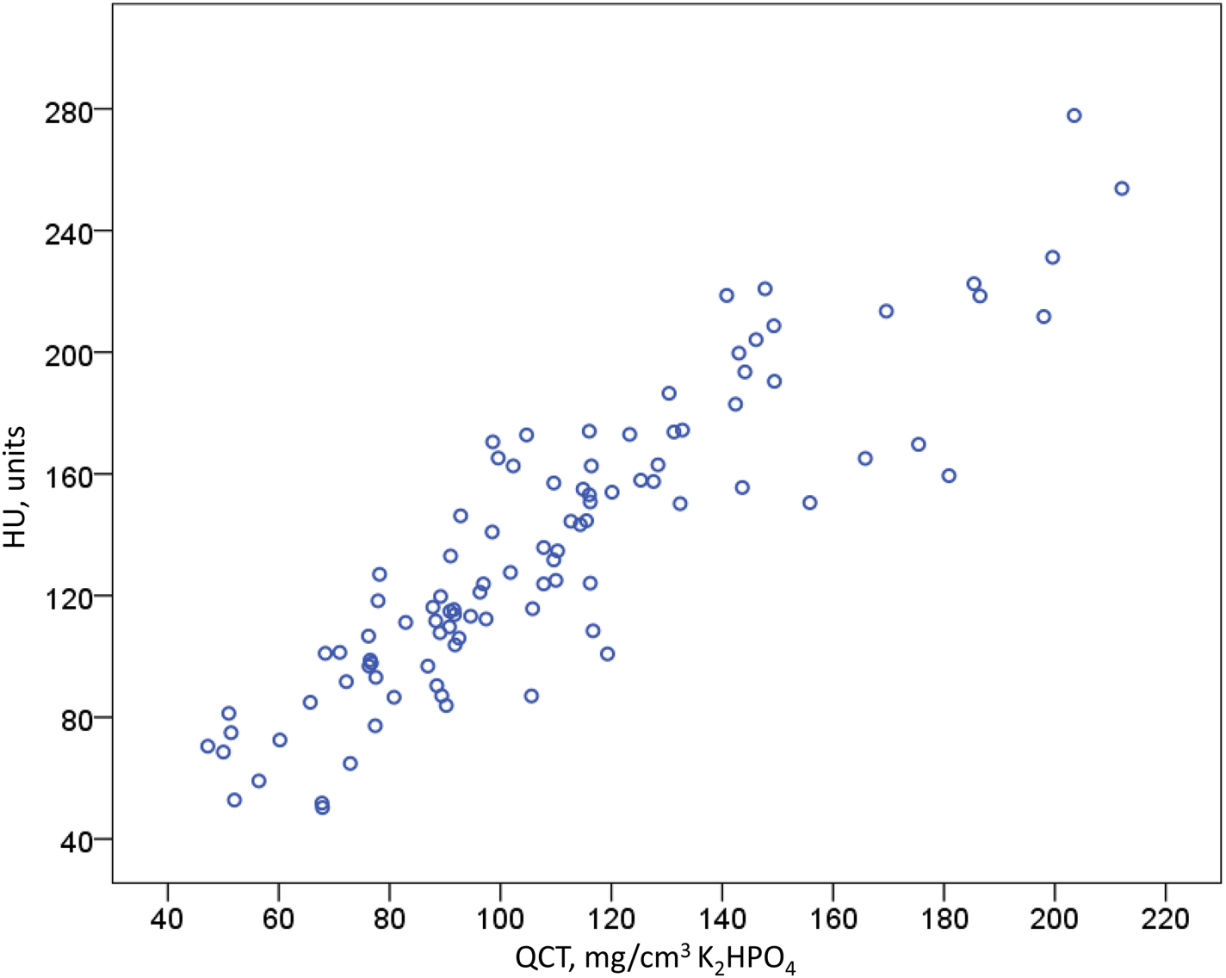
Scatter plot showing quantitative computed tomography values in relation to the mean values of Hounsfield units from the 11th thoracic vertebral body to the 4th lumbar vertebral body, *n* = 98, correlation coefficient *r* = 0.89

**Fig. 3:**
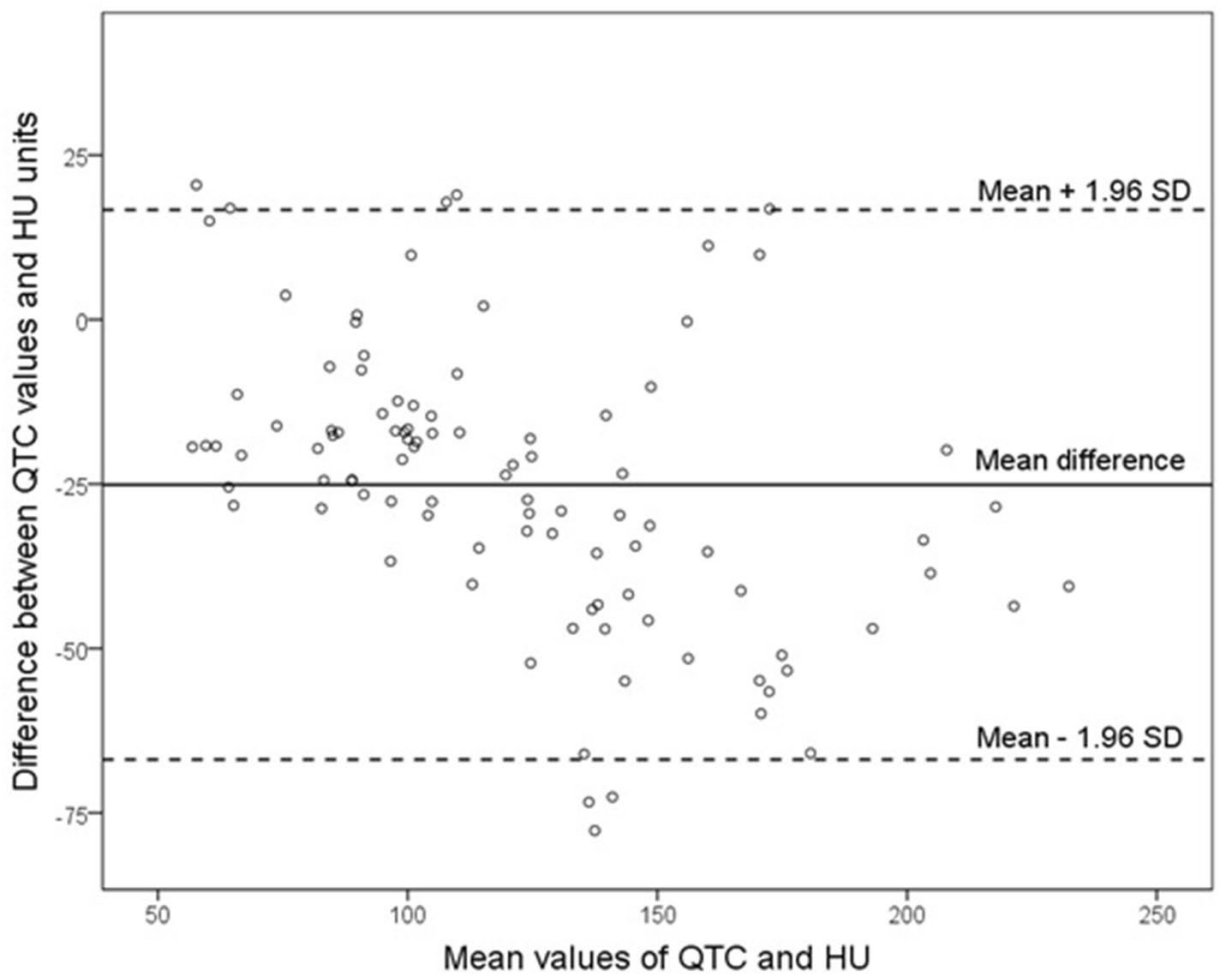
Bland–Altman plot demonstrating the difference between the QTC values and HU: mean: − 25.11; standard deviation 21.32 (1.96 × SD = 41.79, upper limit = 16.68, lower limit: − 66.9)

In the overall comparison, a significant correlation between the HU and the QCT values was found with a Pearson correlation coefficient *r* = 0.894 (*p* < 0.001) (Fig. 2), and this correlation was consistent in both, male (*r* = 0.904, *p* < 0.001) and female patients (*r* = 0.887, *p* < 0.001).Table 2 details correlation coefficients for each vertebral body, and Fig. 3 demonstrates good agreement between the measurements.

**Table 2:** Correlation of the mean values of contrast medium computed tomography and the values of quantitative computed tomography in the individual vertebral bodies

	Count	Correlation coefficient (*r*)^a^	*p* value
Th 11	5	0.823	0.087
Th 12	5	0.900	0.087
L 1	18	0.748	< 0.001
L 2	30	0.902	< 0.001
L 3	25	0.893	< 0.001
L 4	15	0.943	< 0.001
All levels	98	0.894	< 0.001

*L* lumbar, *Th* thoracic

^a^Pearson or Spearman-Rho, as appropriate

### Establishing a conversion formula

Using a logistic linear regression, a following formula was established to approximate bone density values:

QCT-value = 0.71 × HU + 13.82.

## Discussion

The aim of the present study was to examine whether QCT values of vertebral bodies correlate with HU of a contrast medium CT in the thoraco-lumbar spine, to solely rely on contrast medium CT to determine the bone mineral density and to refrain from additional examinations, thus reducing radiation dose.

In our study, we found a strong correlation between HU and QCT values with *r* = 0.894.

Several papers discussing this topic have been published so far [7–10]. However, to the best of our knowledge, our series represents the largest series of vertebrae examined in patients without malignant disease. In most previous studies, no contrast-enhanced CT was used to compare HU with QCT.

In a previous study, Baum et al. examined 15 postmenopausal women who had a QCT and a contrast medium CT [7]. They, too, found a significant positive correlation with a *r* = 0.914.

However, the duration between CT and QCT significantly longer with up to 3 months in their study. In the meantime, changes in bone density may have occurred due to various causes (cortisone therapy, progressive osteoporosis, and advanced tumour disease). To avoid these confounding factors, we only included patients in our study with a very short interval between examinations. The median time between CT and QCT was only 3 days in our cohort.

In another study by Bauer et al. 40 again postmenopausal women were examined with QCT and contrast CT of the abdomen [8]. Here, too, a positive linear correlation with a *r* = 0.98 could be determined. Yet, most patients here received abdominal contrast-enhanced CT to screen for malignant disease, and many suffered from colorectal cancers. Since cancer can also lead to changes in bone density, we chose to analyse patients suffering from trauma and excluded cancer patients.

There are other studies that address this issue. However, in most cases, no contrast medium was given, so these studies are not comparable with our study. The administration of the contrast medium can significantly influence the HU in the CT due to the blood flow, the time of contrast medium administration and the respective tissue. This is often more pronounced in younger patients than in older patients [11, 12]. For example, the contrast medium signal in the area of the Batson venous plexus of the vertebra leads to higher Hounsfield units than in patients who have not received a contrast medium. For this reason, the use of a formula based on a native computed tomography is not transferable to Hounsfield units from contrast agent computed tomographies. In our measurements, we have only included patients who have had a contrast medium CT.

Table 3 lists possible formulas for calculating the bone density value from the various studies as well as our own data. Overall, the studies listed showed similarly high correlations, but the formulas differed by several percentage points in some cases.

**Table 3:** Overview of the different conversion formulas of different authors

Authors, year	Formula
Baum et al. 2012 [7]	QCT-value = 0.695 × HU – 7.9
Bauer et al. 2007 [8]	QCT-value = 0.96 × HU – 20.9
Papadakis et al. 2009 [10]	QCT-value = 0.78 × HU + 10.13
Our data	QCT-value = 0.71 × HU + 13.82

One possible reason could be that only smaller numbers of cases were described in the available studies. In addition, the HU were not determined in multiple planes [8].

It could be possible that the Hounsfield units in contrast medium CT in a single plane do not represent the bone quality of the entire vertebra, as anatomical variants, bone canals or degenerative changes can lead to a deviation in the values. In our work, however, we measured in axial, coronal and sagittal planes.

Deviations in the QCT values may occur due to variations in the position of the patient on the CT table or variability related to different measuring surfaces. This may account for up to 5–15% fluctuations in the measured values with a coefficient of variation of 1.3–1.7% [4, 5, 7, 13]. To minimise these deviations and to achieve valid values, only trained personnel performed measurements in our study in a standardized position using the same technical setting [2, 4, 8, 14].

All these factors can lead to deviations and thus to a low correlation coefficient.

As consequence for daily practice, we use these formula for patients who should operate with screw implantation in the spine. The American College Radiology defines osteoporosis in QCT with a BMD below 80 mg/cm^3^, osteopenia is indicated with a BMD between 80 and 120 mg/cm^3^ and a value above 120 mg/cm^3^ describes a normal bone density [15]. In our clinic, we use perforated screws in patients with a bone density below 120 mg/cm^3^, so that intraoperatively, depending on the screw traction, it can be decided whether the screw should be additionally augmented with cement.

### Limitations

A limitation of our study is its retrospective and monocentric nature. A multicentre study with other CT scanners and protocols would be necessary to strengthen our findings.

All examinations were performed in the context of trauma, but the vertebral bodies involved in the trauma were excluded. However, the patients were not completely healthy.

## Conclusion

Bone density values correlate well to HU measured in contrast medium CT. Using simple formula, the bone density of a contrast medium CT of vertebral bodies can be estimated based on HU without additional examinations and unnecessary costs.
